# Effect of foot core exercises vs ankle proprioceptive neuromuscular facilitation on pain, range of motion, and dynamic balance in individuals with plantar fasciitis: a comparative study

**DOI:** 10.12688/f1000research.136828.1

**Published:** 2023-06-30

**Authors:** Manali Boob, Pratik Phansopkar

**Affiliations:** 1 Musculoskeletal Physiotherapy, Ravi Nair Physiotherapy College, Datta Meghe Institute of Higher Education and Research, Wardha, Maharashtra, 442001, India

**Keywords:** Plantar fasciitis, foot core exercises, ankle proprioceptive neuromuscular facilitation, foot functional index, modified star excursion test.

## Abstract

Plantar fasciitis is generally described as an inflammation of the plantar fascia and adjacent tissues around calcaneus tuberosity. Plantar fasciitis onset has been proposed to have an internal mechanical cause, even though this is inadequately recognized. Studies related to alternation in lower-extremity biomechanics that leads to reduced domes of the foot are hypothesized to cause tension in the underlying fascia. Similarly, despite a wealth of anecdotal data suggesting a direct connection between foot mechanism and disability. This condition typically leads to calf muscular stiffness, soreness in the bottom of the feet, decreased range and foot function, strength, and balance impairment. These results in interference with the normal biomechanics of ambulation. A total of (n=66) individuals with plantar fasciitis will be selected for the trial. Subjects will be allocated to Groups A and B at equal allocation with randomization. Group A will undergo foot core exercises, while Group B will undergo ankle proprioceptive neuromuscular facilitation, with both groups receiving conventional treatment. The regimen lasts for 40 minutes, 5 days, for 6 weeks. The outcome measures will be assessed on Foot Functional Index, modified Star Excursion Balance Test (mSEBT), Visual Analogue Scale (VAS), and Weight Bearing Lunge Test (WBLT) be assessed at the initiation and completion of the entire treatment protocol. Prior and after therapeutic intervention results will be analyzed. Based on the comparison of the two treatments' effects on measuring outcomes in individuals with plantar fasciitis, an analysis will be conducted.

## Introduction

The ankle and foot are constituted of twenty-six separate small bones as well as the major bones of the leg. ^1^ The ankle joint creates a connection that enables the lower extremity to connect with the foot. ^1^ Ankle and foot mobility is described in the following three categorical planes: longitudinal, coronal, and axial. ^2^ The sole of the foot comprises three main vaults: a transverse arch, and two longitudinal arches. The muscles, ligaments, tendons, and fascia in the foot support these domes. ^3^ The gastrocnemius and soleus muscles work together to predominantly plantarflex the ankle. ^4^ The plantar layers contain the intrinsic foot muscles in their anatomical alignment. ^4^ The ends of extrinsic foot muscles are connected at the foot distally and the leg proximally, respectively. ^5^ A broad, longitudinally oriented strand that are firmly linked to the sole of the foot is referred to as plantar fascia. The proximally plantar fascia is attached to the calcaneal tuberosity and distally split into five bands for each toe. ^6^

A gradual degenerative irritation of the plantar fascia, which starts at the medial calcaneal tuberosity, as well as the nearby perifascial tissues, results in plantar fasciitis, an inflammatory disorder. The plantar fascia serves a crucial role in accurate foot mechanics. ^7^ The main prevalent source of heel discomfort is plantar fasciitis. This is responsible for around 15% of all foot issues brought to the attention of healthcare practitioners. ^8^ A mechanical model known as the “windlass mechanism” offers a complete justification for the biomechanical components of the foot. Such knowledge is crucial for clinical practice since it could give medical experts a comprehensive grasp of the connection between pathologies and biomechanical factors. ^9^ A history of pain with the first steps in the morning, pain that becomes worse with loading activities and gets better over the day, and palpable soreness in the medial calcaneal tubercle are used to make the diagnosis of this illness. Achilles tendon tightness may cause patients to have decreased ankle dorsiflexion, which might lead to anticipatory overpronation of the ankle. ^10^

Theories of Proprioceptive Neuromuscular Facilitation (PNF) may be beneficial in enhancing postural balance and stability. In the development of a therapeutic relationship, PNF is characterized as a strategy to improve the neuromuscular stimulation of the proprioceptive sense response mechanism. This technique set contains methods that stimulate proprioceptive sense to improve the response of the neurological and muscular systems. PNF is a stretching method that is utilized in the realms of exercise and sports to increase muscular flexibility, strength, and balance as well as joint mobility. ^11^ The technique emphasis of timing in ankle proprioceptive neuromuscular facilitation (PNF) was enhanced to synchronise and strengthen the movement patterns of both the foot's intrinsic and exterior muscles. ^12^

Exercises for the foot core were done to maintain proper foot alignment, manage the position of the arch, and activate the proprioceptors on the foot's sole to enhance balance. As a result, earlier gait retraining studies have started to include foot core exercise to lower the risk of injury in individuals when the striking pattern abruptly changes. The towel curls, foot doming, and toe spread, which have been shown to be efficient treatments to develop the arch muscles, might be used to strengthen the foot muscles in order to stabilise the form of the arch. ^13^

Visual analogue scale (VAS) is used to assess the pain, rated from zero to ten. The foot functional index (FFI) questionnaire evaluates physical discomfort, impairments, as well as activity-restricting foot pathologies in everyday functioning. ^14^ The 23 items on the questionnaire are categorized into three subdimensions: pain, activity limitations, and disabilities. It was created in English, and it has official translations for usage in Chinese, German, French, Italian, and Spanish. Because standardisation is crucial when utilising measures of assessment, checklists created in other languages must be translated and their parametric qualities assessed to promote comparability between research. ^15^ Ankle dorsiflexion may be directly monitored with weight bearing lung test. The therapist measured the amount of dorsiflexion in affected limbs while the patient will be in a lunge position. The therapists either employed an inclinometer or a measuring tape to evaluate the numerical outcome. In several disorders, including patellofemoral pain syndrome and overpronation of the foot, which may result in plantar fasciitis, it is well-accepted that inadequate ankle dorsiflexion plays a vital role. ^16^

The modified Star Excursion Balance Test is among the popular techniques for the clinical examination of dynamic balance (mSEBT). When assessing the likelihood of lower extremity injuries, clinicians frequently employ just one of these diagnostic techniques and one outcome factor. It is well-established that age, sex, and sport may affect dynamic balance scores. ^17^

The purpose of the study is to compare the effects of foot core exercises and ankle proprioceptive neuromuscular facilitation on pain, range of motion, and dynamic balance in patients with plantar fasciitis. The plantar fascia is subjected to steadily increasing loading stress if these musculatures are weaker and the plantar fascia lacks flexibility, which flattens the longitudinal arch and causes foot eversion. This results in the hip of the afflicted side descending, which causes the hip to rotate inward, and a reduction in the medial joint space of the knee. This may result in plantar fascia overload, muscle imbalance, abnormal gait, and reduced functional performance. Foot core exercises and Proprioceptive neuromuscular facilitation have been shown to be helpful in the reduction of pain, restoring the motion, and regaining muscular strength. There is a lack of literature that emphasizes a comparison between foot core exercises and PNF that helps in improving dynamic state of balance, as well as functional status in patients suffering from plantar fasciitis. As a result, strong evidence is very important in this field.

## Aim and objective

1) To assess and evaluate the effects of foot core exercises on pain (VAS and FFI), range of motion (WBLT), and dynamic balance (mSEBT) in patients with plantar fasciitis.
2) To assess and evaluate the effects of ankle proprioceptive neuromuscular facilitation on pain (VAS and FFI), range of motion (WBLT), and dynamic balance (mSEBT) in patients with plantar fasciitis.
3) To compare the effects of foot core exercises and ankle proprioceptive neuromuscular facilitation on pain (VAS and FFI), range of motion (WBLT), and dynamic balance (mSEBT) in patients with plantar fasciitis.

**Trial Design:** single centric two arm parallel, comparative open labelled superiority trial.

## Protocol

**Figure 1.**
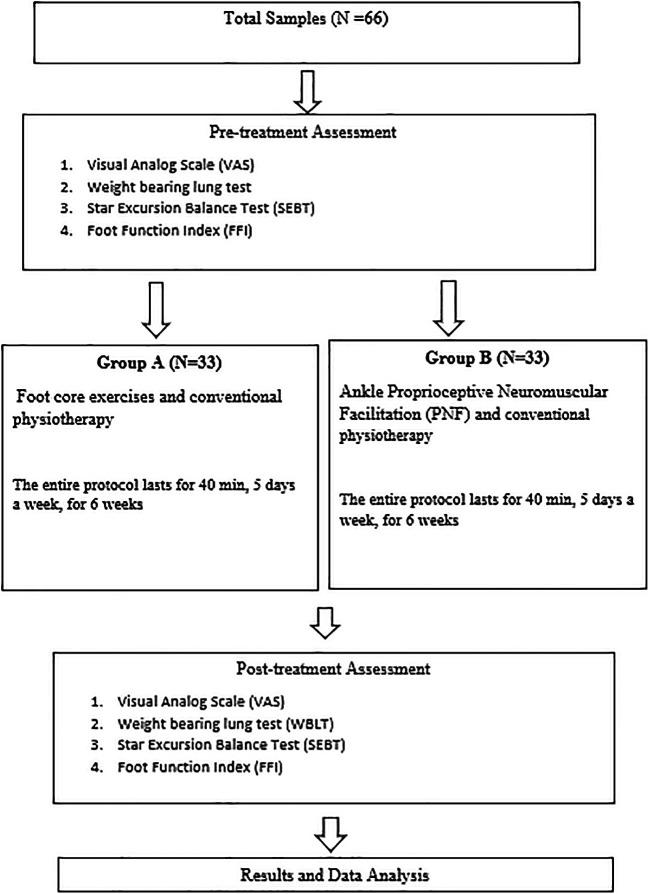
Study procedure flow chart.

The study will be conducted at Musculoskeletal Physiotherapy OPD of Acharya Vinoba Bhave Rural Hospital and Musculoskeletal Physiotherapy OPD of Ravi Nair Physiotherapy college, Sawangi (Meghe) Wardha, after receiving approval from ethical committee of Datta Meghe Institute of Higher Education and Research. The participants diagnosed as plantar fasciitis and follows the inclusion criteria will be randomly selected using simple random sampling and divided into Group A and Group B using computer generated list. After explaining the study protocol in detail, written consent will be signed by the participants. The duration of study will be one year. A total of 33 participants will be in each group. The outcome measures are foot functional index, modified star excursion balance test, weight bearing lunge test and visual analogue scale used to assessed pain, range of motion, dynamic balance, disability, and limitations in activities of daily living. Group A patients will be receiving foot core exercises and conventional therapy and group B patients will be receiving ankle proprioceptive neuromuscular facilitation. The outcome measure will be assessed at the base line and after six weeks of intervention Figure 1. Explains the study procedure.

### Inclusion criteria

1) A progressive onset of heel pain lasting three months or more, localized to the medial calcaneal tubercle.
2) Sharp and worsen pain with the first few steps out of bed in morning.
3) Positive windlass test.
4) Both male and female.
5) Age ranging between 40-60.

### Exclusion criteria

1) Patients with type 1 or 2 Diabetes Mellitus, systemic inflammatory arthritis, cancer, active tuberculosis.
2) Arthrosis of the ankle, knee, and hip.
3) Pregnant women.
4) Patients with numbness or tingling with or without provocation in the lower extremity, undiagnosed pain.
5) Patients currently receiving treatment and using steroids within the last six months for the plantar fasciitis.

### Intervention

**Control group**

Group A will be given foot core exercises which includes heel raise, toe curls, foot doming, toe spreading, balancing board, and tennis ball roll under foot along with conventional therapy which includes ultrasound, plantar fascia stretching and Achilles stretching. The foot core exercises mentioned in Table 1. which lasts for 30 mins with 10 mins of conventional therapy. The entire protocol lasts for 40 min, five days a week, for six weeks.

**Table 1.** Foot core exercises.

Foot core exercises	1-2 weeks	3-4 weeks	5-6 weeks
Double Leg Heel Raise	1 × 10 times	2 × 10 times	2 × 15 times
Single Leg Heel Raise	1 × 10 times	2 × 10 times	2 × 15 times
Towel curls	3 × 10 times	3 × 20 times	3 × 10 times (+0.25 kg)
Foot doming	2 × 10 times	2 × 15 times	2 × 20 times
Toe spread	2 × 10 times	2 × 15 times	2 × 20 times
Balance board	2 × 20 second	2 × 25 second	2 × 30 second
Foot relaxes	1 × 30 second	1 × 30 second	1 × 30 second

**Experimental group**

Group B will be given Ankle Proprioceptive Neuromuscular Facilitation (PNF) which includes techniques such as slow reversal (SR), and rhythmic stabilization (RS) along with conventional therapy. The PNF protocol mentioned in Table 2 which last for 30 mins with 10 mins of conventional therapy. The entire protocol lasts for 40 min, five days a week, for six weeks.

**Table 2.** Ankle PNF technique.

Muscles	PNF technique	Duration (sec) of technique	Repetition of each technique
Ankle Plantarflexors	Slow Reversal	35	3
	Rhythmic Stabilization	90	3
Ankle Dorsiflexors	Slow Reversal	35	3
	Rhythmic Stabilization	90	3
Ankle Invertors	Slow Reversal	35	3
	Rhythmic Stabilization	90	3
Ankle Evertors	Slow Reversal	35	3
	Rhythmic Stabilization	90	3

Legends: proprioceptive neuromuscular facilitation.

### Outcome measures

**Primary outcomes**

1. **Foot Function Index (FFI)**
  the Foot Function Index (FFI) was designed to analyze the effect of foot abnormalities on performance in terms of pain, functional limitations, and activity restriction. The internal consistency was between 0.96 and 0.7. ^15^
2. **The Modified Star Excursion Balance Test (mSEBT)**
  The SEBT (Star Excursion Balance Test) is a multi-dimensional test that includes power, mobility, and postural control. This is a dynamic balancing measurement that poses a substantial challenge to players and healthy and active adults. The evaluation is used to measure functional ability as well as to screen for inadequacies in dynamic balance caused by musculoskeletal disorder. coefficients of variation ranging from 2.0 to 2.9 percent, the mSEBT has been shown to be a valid indicator of dynamic balance. ^17^

**Secondary outcome**

1) **The Visual Analogue Scale (VAS)**
  To determine the intensity of the pain this scale is used. The VAS is a 10 cm line that has two terminals marked with the numbers 0 (“no discomfort”) and 10 (“pain as horrific as it can be”). Ask the patient to rate their current level of discomfort and mark the line accordingly. Using a ruler determine the distance in centimetres between baseline and the current pain mark. ^18^
2) **Weight Bearing Lunge Test (WBLT)**
  Ankle dorsiflexion range is determined with this procedure. In this test with the help of inch tape distance will be measured. Inter-rater ICC values for angles were 0.97 and 0.99. The analysis shows evaluating a dorsiflexion range with this method have great reliability. ^19^

### Sample size calculation

Sample size calculation resulted at 5% level of significance considering Z (1-α) value = 1.96 and (1- β) at power of 80% = 0.84 measuring the expected mean difference for superiority (effect side) of (δ) = 2.602 and standard deviation (σ) = 3.24

Formula Using Mean difference

$$n1=n2=2\frac{{\left(Z_{\alpha }+Z_{\beta }\right)}^{2}\sigma^{2}}{{\left(\delta \right)}^{2}}$$

Primary variable – (Foot Functional Index)

Mean. (Pre) result on Foot Functional Index for foot core exercises with conventional physiotherapy = 72.68

Mean. (Post)- result on Foot Functional Index for foot core exercises with conventional physiotherapy = 59.67 ± 10.69

Difference (mean ± SD) = 13.01 ± 3.24 (As per reference article)

Clinically relevant superiority = 20% = (13.01 *20)/100 = 2.602

$$\text{N1}=2^{*}\left[{\left(1.96+0.84\right)}^{2}\;{\left(3.24\right)}^{2}\right]/{\left(2.602\right)}^{2}=25$$

Minimum samples required = 25 per group

Considering 30% drop out = 8

Total sample size required = 33 per group

Assumptions

$$Z_{\alpha }=1.96$$

α=TypeIerrorat5%

$$Z_{\beta }=0.84\;\left(1-\beta \right)=\text{Power}\;\mathrm{at}\;80\%$$

σ=std.dev

Ref Article: - Jadhav and Gurudut. ^20^

### Analysis

All the results will be calculated using SPSS 27 version. Demographic variables (Confounding factors) as per quantitative assessment will be resulted for finding observational values on mean Standard deviation minimum and maximum. Quantitative assessment will be observed on frequency and percentage. The data for inferential statistics, with outcome variables will be tested for normality using Kolmogorov Smirnov Test. A parametric test will be used if the data follows normal distribution. Non-normal data will be attempted for transformation to normality by using mathematical algorithm such as log or exponential function or box cox method. For finding the significance over the mean pre- and post T-paired will be used for analysis if data still persist with non-normal distribution alternative Wilcoxon test will be used for finding the difference for mean significance between the two groups (Inter) un-paired t-test will be used while the Man-Whitney will be used as an alternative t-test for non-normal data Association and analysis for finding the significance of confounding parameters will be evaluated using Chi square test or Fischer Exact Test or using multi-variant analysis.

### Primary outcome

The two groups will be compared using inferential statistics for their mean change in the primary variable (Foot functional index and modified star excursion test) between baseline and six weeks. For research participants, random effects will be generalised, treatment group and visit count will be taken into consideration while analysing fixed effects. At the conclusion of the intervention, the effect size over the mean change difference on the primary variable will be assessed and the matching 95% confidence interval (CI) given.

### Primary endpoint

To predict the difference in effect size between the active and control groups, secondary outcomes (the visual analogue scale and the weight bearing lunge test) will be examined in accordance with the mixed model effect. If the data is distributed normally, the T-test (unpaired) will be performed to determine whether there is a significant difference between the means of the two groups. The data will be transformed into a normal distribution for non-normal distributions using mathematical techniques. If the primary variable's data still exhibits a non-normal distribution, an alternative non-parametric test will be used (Chi square, Mann Whitney, Wilcoxon test).

### Dissemination

To present the research findings at conferences, seminars, community forums etc.

### Study status

The study is yet to start.

## Discussion

The purpose of this study to analyse the efficacy of ankle pnf vs standard foot core exercises in enhancing functional capacities, enhancing dynamic balance, increasing joint mobility, and reducing discomfort in individuals who are suffering from plantar fasciitis. Poor foot posture can lead to structural abnormalities throughout the body because it places enormous strain on the lower extremity’s joints and muscles. If this abnormal pressure is not alleviated, it will continue to cause discomfort, fatigue, and musculoskeletal injury. When a person has plantar fasciitis, the fascia becomes inflamed because of excessive intense activity, which causes pain and discomfort when doing weight-bearing activities like walking. When engaging in weight-bearing activities, dynamic balance control can be impacted by pain. ^21^

Numerous studies demonstrate the impact on the reduction of pain, improve joint mobility and foot function, etc. According to Ranbhor *et al.,* foam rolling and calf and plantar fascia stretching both aided with pain relief and joint motion. However, the outcome measure shows boosting effects in the foam rolling than the traditional stretching exercises. ^22^ According to Kaur and Koyal's study, stretching the Achilles tendon produced a significant improvement in the patient's outcome variables, but stretching the calves reduced discomfort and foot function deficit but did not have the same impact. ^23^ According to Kumar *et al*., low dye taping combined with stretching of the plantar fascia and strengthening of the intrinsic muscles was an effective method for reducing pain and enhancing functional activities. Both groups showed a substantial improvement in all categories, however the people who had low dye taping saw a considerably superior overall improvement. Consequently, low dye tape is an effective intervention method for the treatment of plantar fasciitis patients. ^24^

By analysing the outcome, we will be evaluating the impact of foot core exercises and ankle pnf on dynamic balance and compare both the intervention in this study. Muscle strength in the fundamental body parts is crucial for maintaining balance. Accurate and efficient exercise of these muscles appears to be of utmost importance since they play a significant function secondary to that of the foot, as well as in relation to the complete kinematic chain.

### Ethical considerations

Institutional Ethics Committee of Datta Meghe Institute of Higher Education and Research (Deemed to be University) approved this study.

DMIHER (DU)/IEC/2023/804 is the reference number.

## Data Availability

Not applicable as this is a protocol. Zenodo: SPIRIT Checklist DOI: 10.5281/zenodo.7990088. ^25^ Data are available under the terms of the Creative Commons Attribution 4.0 International license (CC-BY 4.0).
