# Supplementary material for: Effect of foot core exercises vs ankle proprioceptive neuromuscular facilitation on pain, range of motion, and dynamic balance in individuals with plantar fasciitis: a comparative study
Source: F1000Res. 2023 Jun 30;12:765. [Version 1] doi: 10.12688/f1000research.136828.1 (PMC11826074; doi:10.12688/f1000research.136828.1)
Supplement: Peer review history

# Reviewer report: Reviewer response for version 1

**DOI:** 10.5256/f1000research.149973.r234132

**Published:** 2024-02-20

**Authors:** Elif Tuğçe Çil

**Affiliations:** 1 Department of Physiotherapy and Rehabilitation, Yeditepe University, Istanbul, Turkey

Thanks to the authors for their efforts.

 -The terminology of plantar fasciitis should be explained in an up-to-date manner. Because this condition is not only associated with inflammation. 

 -The discussion section should be written by discussing and interpreting the current literature in comparison with your results. 

 -The language of the article contains many grammatical errors and should be corrected. 

 -I did not see any table of your result.

Is the study design appropriate for the research question?

Yes

Is the rationale for, and objectives of, the study clearly described?

Yes

Are sufficient details of the methods provided to allow replication by others?

Yes

Are the datasets clearly presented in a useable and accessible format?

No

Reviewer Expertise:

Physiotehrapy

I confirm that I have read this submission and believe that I have an appropriate level of expertise to confirm that it is of an acceptable scientific standard, however I have significant reservations, as outlined above.

# Reviewer report: Reviewer response for version 1

**DOI:** 10.5256/f1000research.149973.r209275

**Published:** 2023-10-02

**Authors:** Pornpimol Muanjai

**Affiliations:** 1 Burapha University, Chonburi, Thailand

Introduction

- The rational in term of using these two intervention is still weak. Please provide more the beneficial effects to plantar fasciitis condition. 
- "PNF is a stretching method.." It sounds a bit wrong. Please kindly check or rephrase as it is a kind of neuromuscular facilitation acts as muscle contracting or stretched.
- Fifth paragraph can be shortened regrading available languages.
- Please explain more why plantar fasciitis affects to balance deficit.
- The study hypothesis should be given.
- References needs for "foot core exercises and PNF have been shown to be helpful in..." 

Methods

- Please provide the ethical approve number for this study.
- Please specify in case bilateral foot involved, which side will be trained and tested.
- Please specify the treatment detail where those received and will be excluded from the study.
- Why those 2 PNF technique were selected in this study? It would be useful to know for the other.
- Please reconsider the foot core exercise program as in the review it was suggested that those 3 exercises were effective treatment for arch correction. Personally, I would suggest to remove the double leg heel raise as single one would be more loading and also the tennis ball rolls which it should be as a part of the conventional treatment for plantar fascia stretching for both groups.
- What activity VAS will be examined? at rest or walking?

Discussion

- It would be better if the evidence regarding the using these 2 interventions in plantar fasciitis or on those parameters will be more available. 

There was no data to report yet.

Is the study design appropriate for the research question?

Yes

Is the rationale for, and objectives of, the study clearly described?

Partly

Are sufficient details of the methods provided to allow replication by others?

Partly

Are the datasets clearly presented in a useable and accessible format?

Not applicable

Reviewer Expertise:

Exercise, aging, physical therapy

I confirm that I have read this submission and believe that I have an appropriate level of expertise to confirm that it is of an acceptable scientific standard, however I have significant reservations, as outlined above.
